# Motor and non-motor outcomes post rehabilitation for persons with freezing of gait and Parkinson's disease: A systematic review and Meta-analysis

**DOI:** 10.1016/j.prdoa.2026.100498

**Published:** 2026-09-07

**Authors:** Jessica E. Bath, Diane D. Allen, Valerie J. Block

**Affiliations:** a University of California, San Francisco, Department of Physical Therapy and Rehabilitation Science, USA; b Department of Physical Therapy, San Francisco State University

**Keywords:** Parkinson's disease, Rehabilitation, Freezing of gait, Cognition, Mood, Postural control

## Abstract

**Introduction:**

Freezing of gait (FOG), a symptom of Parkinson's disease (PD), involves dysfunction across motor, cognitive, and limbic domains. Rehabilitation for PD traditionally emphasizes motor impairments, leaving non-motor FOG contributors underexplored, which may contribute to limited understanding of mechanisms underlying FOG or optimal FOG rehabilitation. To investigate this, we quantified within-group clinical outcomes related to postural control, executive function, and limbic domains to evaluate the impacts of rehabilitation studies on multidomain FOG pathophysiology in people with PD and FOG (PD + FOG).

**Methods:**

Search of PubMed and CINAHL used terms PD, FOG, rehabilitation, postural control, cognitive, and limbic outcomes. Full-text articles were assessed by two reviewers to confirm their eligibility. Eligible studies utilized >1 rehabilitation intervention session, reported pre- and post-training data for ≥1 FOG-related symptom domain outcome specifically for PD + FOG. Fixed or random effect meta-analyses were conducted for each domain, resulting in summary effect sizes Cohen's d (95% confidence interval) reflecting standardized within-group changes.

**Results:**

The search returned eighteen studies eligible for data extraction. Postural control results: 17 unique participant groups (*n* = 458), *d* = 0.49 (0.36, 0.62). Cognitive processing results: 8 unique participant groups (*n* = 193), d = −0.22 (−0.42, −0.02). Limbic (anxiety, depression) results: 4 unique participant groups (*n* = 55), d = −0.08 (−0.45, 0.30).

**Discussion:**

Postural control and cognitive domain outcomes demonstrated significant post-intervention improvements following rehabilitation for PD + FOG. Limbic domain results showed small, nonsignificant pre-post changes. Few studies reported clinical outcome measures covering motor and non-motor domains. Further studies should assess motor and non-motor FOG-related domains and more comprehensive rehabilitation approaches for PD + FOG populations.

## Highlights

• Freezing of gait is a multidomain phenomenon (motor, cognitive, limbic).
• Rehabilitation of freezing of gait remains focused on addressing motor impairments.
• Our review and meta-analysis synthesized multidomain rehabilitation outcomes.
• Postural control improved, outcomes less clear for cognitive and limbic outcomes.
• Findings highlight mismatch between the pathophysiology of FOG and rehabilitation.

## 1 Introduction

### 1.1 Background

Freezing of gait (FOG) remains a highly disabling and refractory symptom of Parkinson's disease (PD), ultimately affecting up to 80% of the 1.1 million people living with PD in the United States [11], [18]. Defined as the brief, episodic stoppage of walking despite the intention to walk, FOG leads to significantly increased fall rates in people with PD and FOG (PD + FOG), as well as decreased mood and quality of life [34], [38]. Despite its prevalence and burden, FOG's complex and multidomain neural mechanisms hinder the identification and implementation of effective medical interventions, including rehabilitation and PD-specific exercise [32].

Converging research [3], [14], [32] indicates that FOG arises from dysfunction across overlapping basal ganglia-thalamo-cortical circuits. This dysfunction produces associated impairments which can variably span limbic [15], [25], [55], cognitive [15], and motor [38] “domains” in PD + FOG. For example, individuals with FOG often report higher anxiety [55], while depression symptoms tend to increase the risk of FOG [25]. These limbic symptoms may reflect dysfunctional signaling occurring between the cortex, striatum, and amygdala, which alters the threat response and contributes to motor breakdown [20]. FOG is also associated with executive dysfunction thought to stem from abnormal signaling in frontal-basal ganglia circuits, specifically when shifting between activities (cognitive flexibility) or preventing an undesired response (cognitive interference) [13]. Further, the limbic and cognitive domains tend to interact with the postural control (motor domain) aspects: slower processing speed in PD + FOG is linked to prolonged and unstable postural preparation, altering gait initiation and reactive balance responses [13]. Despite this multidomain conceptualization and pathophysiology, rehabilitation and PD-specific exercise for FOG and its outcomes remain largely focused on addressing motor domain impairments.

The clinical practice guideline (CPG) for PD rehabilitation strongly recommends gait training with external cueing, balance training to improve postural control, and community-based exercise [39]. Although these approaches are often effective for improving motor disease severity and gait-related metrics, data remain limited for populations with advanced disease or greater fall risk such as PD + FOG [39]. The CPG also strongly recommends the use of integrated care methods for PD, however, only 3 of the 16 studies analyzed included non-motor outcomes such as anxiety or depression [39]. A focus on motor domain impairments is expected to limit the extent to which rehabilitation and exercise address prominent non-motor FOG symptoms and broader disability.

Delivering effective, comprehensive rehabilitation for PD + FOG is complicated by limitations of common clinical measures assessing FOG. The Freezing of Gait Questionnaire (FOG-Q) is widely used to capture an individual's reported FOG frequency and severity and differentiate those with FOG [49]. However, it does not survey one's FOG triggers or the relative contribution of motor, cognitive, or limbic aspects of FOG that influence dysfunction. The questionnaire also demonstrates poor sensitivity [27], limiting its assessment of treatment-related changes. More comprehensive insights could further FOG-related mechanistic insights, while greater assessment specificity across motor and non-motor FOG-related domains could help characterize symptom responsiveness and inform individualized PD + FOG rehabilitation.

Literature specific to PD + FOG rehabilitation has revealed several gaps. Many clinical trials focus on motor-domain training only. Some studies use the FOG-Q (or New-FOGQ) as the primary outcome, rather than continuous, objective measurements of motor performance or changes in the cognitive and limbic domains [21]. A meta-analysis suggested that rehabilitation specifically targeting the “underlying correlates of FOG” may be more effective than generic rehabilitation for PD, but few studies have directly compared the effects of different types of training for this population [21]. Some existing studies include mixed or ambiguously-characterized PD cohorts with and without FOG [21], limiting the specificity and clinical translation of results. Lack of data on PD + FOG exclusively, multi-domain impairments, and specific outcomes of interest may contribute to the often-prolonged episodes of rehabilitative care (e.g. 40–90 min/session, 2–3 times/week, for up to 6 months) associated with meaningful benefit for this population [30].

Recent advances have begun to acknowledge and address these challenges. The Characterizing Freezing of Gait Questionnaire (C-FOGQ) was developed and validated to assess limbic, cognitive, and motor FOG-related issues in PD + FOG, however, has not been adopted in clinical settings [16]. A recent intervention study featured a multi-domain treatment approach in PD + FOG. Fifteen participants completed discrete 4-week training blocks each targeting the limbic, cognitive, and sensorimotor FOG-related symptom domains [9]. The primary outcomes were FOG-related metrics rather than domain-specific clinical outcome measures, however. While the authors reported mixed results, individualized and/or combined therapeutic approaches across domains were recommended [9].

To address some of these gaps, we conducted a systematic review and meta-analysis of specific motor and non-motor outcomes following PD + FOG rehabilitation. We synthesized multi-session (>1) rehabilitation studies in PD + FOG that reported clinical outcomes reflective of motor (postural control), cognitive (executive function), or limbic (anxiety and/or depression) FOG-related domains. Within-group treatment effects were quantified for each domain to capture the capacity for domain-specific rehabilitation-related changes. We expected that rehabilitation would produce significant improvements across all domains in PD + FOG, however, anticipated that the quality of evidence and number of studies for the limbic and cognitive FOG-related domains would be limited compared to the motor domain. We expected our findings to help guide rehabilitation for the multi-domain pathophysiology of FOG.

## 2 Methods

### 2.1 Inclusion/exclusion criteria

Studies were included based on pre-established criteria: (i) prospectively collected data regarding the effects of a skilled exercise or rehabilitation-based intervention spanning >1 training session; (ii) reported clinical outcomes reflective of ≥1 FOG-related symptom domain (motor, cognitive, limbic); (iii) had a full retrievable English text; and (iv) had extractable data from participants with diagnosed PD and self-reported or documented FOG. Exclusion criteria included: (i) case study or case series, conference abstract, or review articles; (ii) the intervention consisted of general health education (not FOG-specific); (iii) the intervention consisted of 1 session (assessing same-day effects); (iv) the intervention featured invasive or non-invasive stimulation-based interventions alone or with training (e.g., deep brain stimulation, peripheral electrical stimulation), or (vi) the training consisted solely of walking with or without external cueing (visual, auditory, tactile), without additional skilled and structured therapeutic elements, such as progression with established criteria, motor learning strategies, dual-task integration, biofeedback, etc.

### 2.2 Outcome measures

Selected outcomes addressed the three FOG-related symptom domains with validated clinical tests assessing impairments in PD + FOG: Cognitive measures (reflecting executive function, specifically attentional set-shifting and processing speed) included the Stroop test [52] or Trail-Making-Test Part B [51]; and Limbic measures (reflecting anxiety and/or depression) included the Hospital Anxiety and Depression Scale [35], Depression, Anxiety, and Stress Scale [28], Geriatric Depression Scale [17], or State Trait Anxiety Inventory [31]. Motor measures (reflecting postural control) used the Mini Balance Systems Evaluation Test [23], Berg Balance Scale [44], Fullerton Advanced Balance scale [46], or the Gait and Balance Scale [53].

### 2.3 Search strategy, databases, and search terms

The study followed the Preferred Reporting Items for Systematic Reviews and Meta-Analysis (PRISMA) checklist [40]. Two relevant databases (PubMed and CINAHL) were searched without filters from study conception (December 2025) until February 17th, 2026. The search was repeated on May 4th, 2026; duplicates were removed using the software Covidence (Veritas Health Innovation, Melbourne, Australia). Search terms included Parkinson's disease and freezing of gait and derivatives, as well as various rehabilitation or exercise-based interventions (e.g., Action Observation Training, mindfulness, Tai-Chi), and common clinical outcomes reflecting motor (postural control), cognitive (e.g., inhibition, cognitive flexibility) and limbic (mood) domains (Supplemental Material #1). To assist with the initial generation of candidate search terms and Boolean syntax, Chat GPT (OpenAI) was used. The authors subsequently reviewed, modified, and finalized all search terms and strategies prior to use.

### 2.4 Study selection

Two authors (J.B., V.J.B.) independently searched the databases and screened non-duplicate results. Studies were first screened by title and abstract to assess their general appropriateness to the research question. Remaining studies were screened for eligibility using the pre-set criteria above. In the event of a discrepancy in eligibility decisions, the authors discussed and reached a consensus. Covidence was utilized to assist with search organization and screening.

### 2.5 Quality assessment and risk of bias

Risk of bias and study quality were addressed using PEDro (Physiotherapy Evidence Database) [7] and STROBE (Strengthening the Reporting of Observational studies in Epidemiology) guidelines [12]. GRADE (Grades of Recommendation, Assessment, Development and Evaluation) was used to determine the strength of the recommendations from this meta-analysis [24]. Bias and study quality were assessed by authors J.B. and V.J.B., with consensus reached via discussion.

### 2.6 Data collection and synthesis

Within-group meta-analyses were performed for the cognitive, limbic, and motor domains. Data were extracted as pre- and post-intervention means and standard deviations. Data given as standard error instead of standard deviation or geometric means instead of arithmetic means were converted as appropriate in accordance with published methods [26]. For one study [4], summary data were not provided for the selected outcome measures, so data were extracted from figures by two authors (J.B. and D.A.) independently with consensus reached upon discussion. These authors (J.B. and D.A.) performed all data extraction separately, with discussion and consensus reached if differences arose.

Individual within-group standardized mean differences (Cohen's *d)* with 95% confidence intervals (CI) were calculated to reflect standardized changes from studies within each domain. Effect sizes were weighted by inverse variance and pooled within each domain to generate a grand effect size (CI). Heterogeneity across articles was assessed using the Q heterogeneity statistic. A significant Q (*p* < 0.05) led to use of a random effects model for the meta-analysis; if Q was not significant, a fixed effect model was used.

Conversion of pooled effect sizes back to original clinical units was performed for each FOG-related symptom domain to facilitate interpretation and comparison with established minimal clinically important difference (MCID) values where available. Between-group analyses were not performed due to substantial heterogeneity in comparator groups and limited control group data. Consequently, our results should be interpreted as rehabilitation-associated findings across FOG-related symptom domains, rather than synthesized estimates of comparative treatment efficacy for the included outcome measures.

## 3 Results

### 3.1 Study selection

**Fig. 1:**
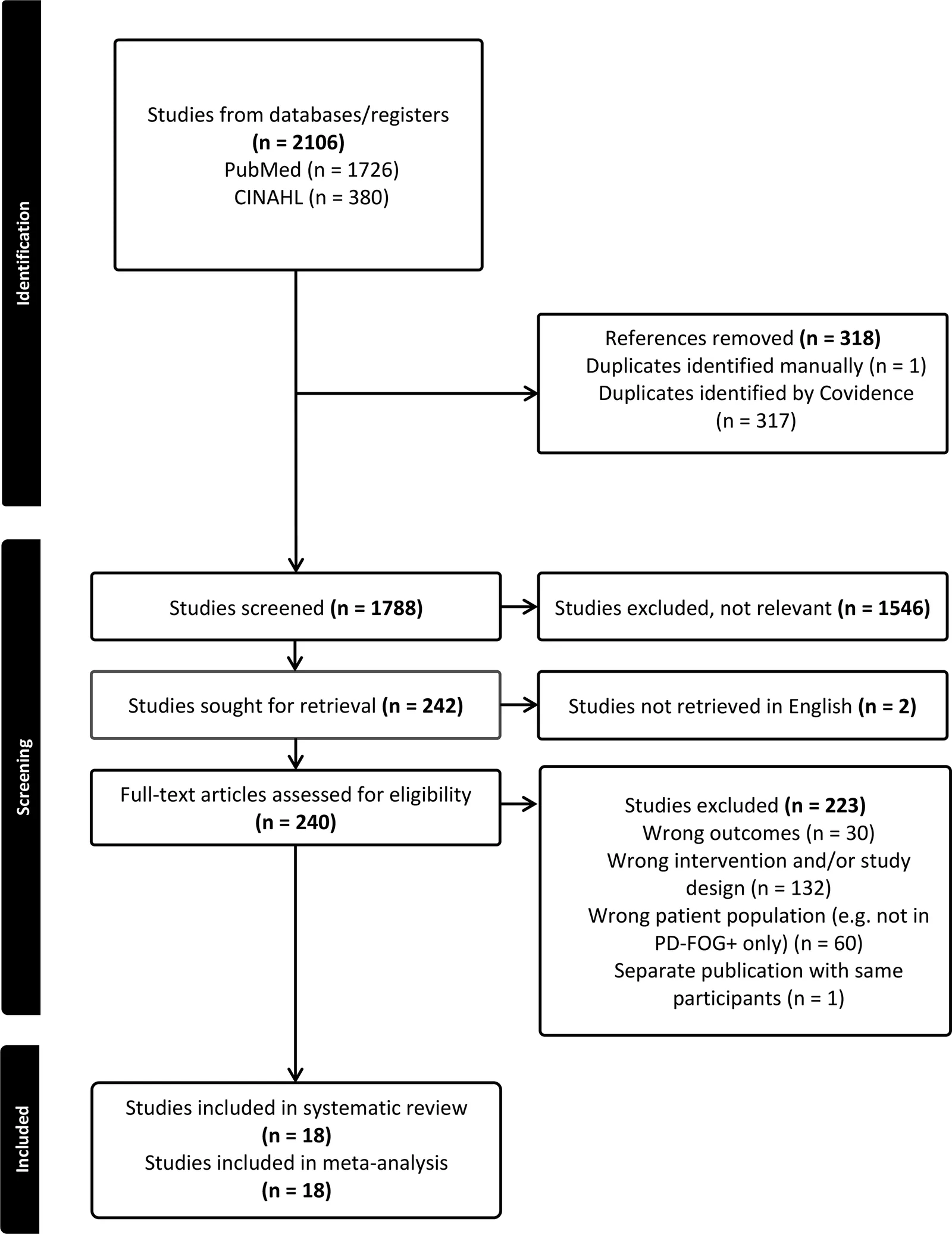
PRISMA Diagram.

The search returned 2106 records from two databases (PubMed *n* = 1726; CINAHL *n* = 380). With duplicate studies removed, 1788 studies remained and were screened by title and abstract. Following screening, 240 full-text articles were assessed for eligibility. Eighteen studies remained and were included in meta-analysis. The selection process is provided in a PRISMA diagram (Fig. 1). Prior to submission, the search was repeated, and 36 additional articles were retrieved; however, none met criteria for inclusion.

### 3.2 Primary study summary

#### 3.2.1 FOG-related motor domain

Twelve studies provided within-group data reflecting postural control following rehabilitation in PD + FOG. Of these, five studies provided data from two eligible intervention arms with separate participant groups. Thus, 17 unique participant groups and interventions were included, totaling 458 PD + FOG individuals (Table 1).

**Table 1:** Study Summary.

Study, authors	Study Design, Quality of Evidence	Population	Intervention	Intervention Parameters	FOG-Related Domain(s), Outcome(s)	Findings on FOG-Related Domain(s); Data Extraction Notes	Harm and/or Cost
Agosta et al. (2017) [1]	RCT with experimental (Action Observation Training) or active control Quality: Good (6/10; PEDro scale)	PD + FOG (*n* = 11 for AOT group)	In the AOT group, participants watched videos of strategies such as weight shifting, walking with long steps, turning, etc. for circumventing FOG and imitated the movements with auditory cues	Training was 60 min. Over 3×/week for 4 weeks Delivered by a PT, unclear whether 1–1 or group-based delivery	Motor Domain, Berg Balance Scale (BBS)	For the AOT group, BBS increased from 50.9 +/− 3.8 to 53.6 +/− 2.6; *p* = 0.002	None reported
Ashburn et al., 2019 [2]	Design: 2-group, multicenter, RCT Quality: Good (8/10; PEDro scale)	PD living at home with ≥1 fall. Subgroup analyses performed for those PD + FOG (*n* = 110)	Progressive balance, strengthening, and general fall avoidance education & training for fall reduction	Training mean = 11 sessions. Frequency: Either 2/week for 3 weeks, then 1×/week for 1 week or 2×/week for 2 weeks, then 1×/week for 2 weeks Delivered 1–1 by a PT, in person with home exercises & DVDs for daily performance	Motor Domain, MiniBEST	Statistically significant improvement on MiniBEST for PD + FOG at 6mos compared to controls (*p* = 0.017); Change from baseline mean (sd): 1.4 (3.6)	Extensive cost analysis: program for PD + FOG not cost-effective Harm: SAE reported but none related to study
Bekkers et al., (2020) [4] **(Dual-arm)**	Multi-site RCT with subset analysis Participants assigned to treadmill training + virtual reality (TT + VR) or skilled treadmill training (TT) Quality: Good (6/10; PEDro checklist)	PD + FOG subset (total *n* = 77) VR + TT: *n* = 34 TT: *n* = 43	Treadmill (gait) training with progressive increases in gait and walking duration +VR group: Obstacles added & progressed for increased height/depth, change in visibility, busyness, etc.	Training was performed 3×/week over 6 weeks; 45 min. Each session Program was supervised by PTs trained in the study's delivery methods and fidelity checks performed	Motor Domain, MiniBESTest Cognitive Domain: Trail-Making-Test Part B (TMT-B)	MiniBESTest: VR + TT: Improved from mean (sd): ∼21.5 (4.66) to ∼23 (4.66) TT: Improved from ∼20.5 (3.28) to ∼22 (3.28) TMT-B times: VR + TT: Decreased from ∼155 s (70) to 133 s (58.3) TT: Decreased from 165 s (81.97) to 160 s (81.97) *Point estimates for extraction estimated from line plots*	None reported
Brewer Mixon et al., (2025) [5]	Pilot, single-arm clinical trial Quality: Excellent (20/22, 91%; STROBE checklist)	PD + FOG (*n* = 10)	The intervention utilized cognitive behavioral therapy (CBT). The first 2 sessions were spent developing rapport and a thought record, with the second 2 sessions covering mindfulness and application	Intervention consisted of 4 weekly individual intervention sessions delivered by a neuropsychologist	Limbic Domain, The Depression Anxiety Stress Scale-21 (DASS-21): Anxiety subscore only	The DASS-Anxiety subscore improved (decreased) from baseline: mean (sd): 6.6 (4.22) to post-testing: 3.9 (2.77); *p* = 0.0775	None reported
Capato et al., (2020) [6] **(Dual-arm)**	Single-blind RCT; post-hoc analysis 3 study arms: 1) Multimodal balance training with RAS; 2) Multimodal balance training, no RAS; 3) Education group (not included in meta-analysis) Quality: Good (8/10 PEDro scale)	PD + FOG sub-analysis: (total *n* = 37) + RAS: *n* = 19 -RAS: *n* = 18	Multimodal balance training: Gait & balance training (40 exercises, e.g. multidirectional stepping, reactive balance, agility training etc.) + RAS: Open-loop auditory cueing via individually-set metronome during balance training	Training consisted of 10 sessions of 45 min. Each (2×/week for 5 weeks) Progressive program; led by PT	Motor Domain; Mini-BESTest	*PD + FOG results only:* + RAS: MiniBESTest improved from mean (95% CI): 11.1 (7.8–14.3) to 18.4 (15.3–21.5) -RAS: MiniBESTest improved from mean (SE): 11.7 (8.2–15.2) to 14.7 (10.8–18.4) + RAS to control: *p* < 0.001 - RAS to control: *p* = 0.011	None reported
Chen et al., (2023) [8] **(Dual-arm)**	RCT; “Experiment #2” data only 2 study arms: Training with internal or external cueing Quality: Good (7/10 PEDro scale)	PD + FOG (total *n* = 30) Internal focus group: *n* = 15 External focus group: n = 15	Dual-task walking under varied conditions (e.g. stepping, around obstacles, narrow spaces) Secondary task: added cognitive and motor tasks with cued internal or external focus while walking	Training consisted of 2 sessions/week for 6 weeks (total n sessions =12) Warm up 5 min, dual-task walking 50 min., cool-down 5 min. Physical therapist led training; unsure of further delivery details	Motor Domain, Berg Balance Scale (BBS)	Internal focus group (ON med): The BBS score improved from mean (+/− SE): 49.40 +/− 1.69 to 53.40 +/− 0.53 (*p* < 0.01) External focus group (ON med): The BBS score improved from mean (+/− SE): 49.80 +/− 1.10 to 52.40 +/− 0.82 (p < 0.01).	Harm: Authors reported “all participants completed the experiment without adverse events and falling”
Clerici et al., 2019 [10] **(Dual-arm)**	Single-blind, parallel group randomized trial Data extracted from 2 (active) study arms with separate participant groups Quality: Good (7/10; PEDro scale)	PD + FOG (total *n* = 52) MIRT (*n* = 25) MIRT+AT (*n* = 27)	MIRT: Multidisciplinary, aerobic, intensive rehabilitation treatment for PD (inpatient) MIRT-AT: Land-based MIRT +3 sessions per week of aquatic therapy Balance/gait training, OT, SLP; psychoeducation	Training: 4 weeks in a hospital setting with 4 daily training sessions for 5 days; 6th day has 1 h of aerobic exercise. Psychoeducational therapy 1 h per week for 4 weeks Optional robotic-assisted walking or VR training (3 × 1-h sessions for 3 weeks)	Motor Domain, Berg Balance Scale (BBS)	MIRT: BBS improved from (mean (sd): 43.2 (9.3) to 50.8 (5.6) MIRT-AT: BBS improved from (mean (sd): 45.7 (7.4) to 53.0 (3.4) p (time) for both programs ≤0.0001	Harm: 2 participants in the MIRT+ AT group dropped out from “discomfort” in the water Cost: The “expensive nature” of treatment was mentioned.
Frazzitta et al., (2013) [19]	Single-arm clinical trial with sub-analysis for PD-FOG+ Quality: Fair (14/22, 64%; STROBE checklist)	PD + FOG (total *n* = 30) 1, 2,	Progressive treadmill (gait) training with visual and auditory cues; feedback if pt.'s footfalls were within individualized targets	Training was performed for 30 min., 5 days/week for 4 weeks (20 total sessions)	Motor Domain, Berg Balance Scale (BBS)	The BBS score improved from mean (+/− sd): 43.3 +/− 8.4 to 51.3 (change score given only: 8.0); *p* < 0.0001 (time x group)	None reported
King et al., (2020) [29]	Randomized, single-blinded crossover study Quality: Fair (4/10; PEDro scale)	PD + FOG (*n* = 42)	Progressive circuit-based exercises, including gait & functional training, agility obstacle course, lunges, boxing, adaptive tai-chi +/− dual-tasking	Training was 80 min. Exercise sessions performed in groups (2–6 people/group) for 3d/week for 6 weeks. Delivered by an exercise trainer with oversight from a PT and aide from trained research assistants	Motor Domain, MiniBEST Cognitive Domain, Stroop Interference (ratio of naming colors/incongruent colors-words)	Mean (sd) improvement following exercise: −0.15 (0.7) Standardized response mean (95% CI): 0.22 (−0.12–0.55) *Data extracted pre/post exercise intervention only. (“Education” program did not meet study inclusion criteria)*	Harm: 1 participant fell during a class, resulting in a hip fracture & 4 non-injurious falls occurred in classes
Mezzarobba et al., (2018) [36] **(Dual-arm)**	Pilot RCT (2 active participant groups) Quality: Good (7/10; PEDro scale)	PD + FOG (total *n* = 22) Action Observation Training + sonification AOS): *n* = 12 AOT + cueing: *n* = 10	Participants watched 8 videos showing different motor gestures (e.g. weight shifting, step coordination), then asked to repeat the movements using videos composed by the images & sounds of the gestures & PT feedback The AOT + cueing group utilized external cues w/o sonification	Training consisted of 1 h. 2×/week for 8 weeks (total of 16 training sessions) led by a PT	Cognitive Domain: Trail-Making-Test Part B (TMT-B) (s)	AOS: The TMT-B time increased from mean +/− sd: 147.34 s +/− 116.51 to 148.35 +/− 199.33 AOT with cueing: The TMT-B time decreased from mean +/− sd: 190.58 s +/− 130.99 to 166.68 s ±117.38 *Data were extracted from 1-month post intervention follow-up (no post-test data generated)*	Harm: 2 (unrelated) medical events caused 2 participants to drop out following enrollment
Nuic et al., (2018) [37]	Pilot feasibility study; single-arm Quality: Good (16/21, 76%; STROBE checklist)	PD + FOG (n = 10)	Custom videogame with Kinect motion sensor for biofeedback Participants performed large amplitude movements (e.g. weight shifts, flexion/extension) while controlling avatar	Training consisted of 18 sessions across 6 weeks Participants' within-session training dose progressively increased from 15 to 43 min (207–932 movements) across the intervention duration	Motor Domain, Gait and Balance Scale, Part B (GABS-B)	The GABS-B decreased from mean (sd): ∼18 (9) to ∼12 (8); *p* < 0.05) *Point estimates for extraction estimated from boxplots with author agreement; sign changed to match directionality of other outcome measures*	Harm: 3 serious adverse events reported but unrelated to training; “no specific adverse events”
Pelosin et al., (2018) [41]	RCT with experimental group (Action Observation Training, AOT) or active control Quality: Fair (5/10; PEDro scale)	PD + FOG (*n* = 32 for AOT group)	Participants watched videos of strategies such as weight shifting, walking with long steps, turning, etc. for circumventing FOG and imitated the movements	Training was 2×/week for 5 weeks, each session lasted 45 min. Delivered by a PT in a small group setting	Motor Domain, Berg Balance Scale (BBS)	For the AOT group, the BBS improved from mean (sd): 46.3 +/− 8.5 to 51.3 +/− 5.7 (*p* < 0.001)	None reported
Plotnik et al., (2014) [42]	Pilot, single-arm clinical trial Quality: Good (15/21, 71%; STROBE checklist)	PD + FOG+ (*n* = 15)	The intervention consisted of progressive motor learning training with FOG-provoking situations and RAS cueing with desired outcomes such as lengthened step and rhythmicity Cognitive and daily living tasks were added over time & combined with training	Training was carried out for 3×/week for 6 weeks Training location was varied from the gait laboratory to gradually more busy/realistic environments	Limbic Domain, Geriatric Depression Scale (GDS)	GDS worsened following the intervention, from mean +/− SEM: 3.5 +/− 0.8 to 3.9 +/− 0.8 (*p* = 0.567)	Harm: No adverse events reported or issues with exhaustion or fatigue.
Rawson et al., (2018) [45]	Pilot feasibility study; single-arm Quality: Excellent (19/21, 90%;STROBE checklist)	PD + FOG (*n* = 7)	Classes included FOG education/discussion, practice of strategies,& goal-setting & review related to skill practice at home; caregivers allowed to listen & observe training	Training consisted of an intensive community-based “bootcamp” for PD + FOG Program lasted 6 weeks, with 1.5-h classes each week designed and taught by two PTs	Motor Domain, MiniBESTest	MiniBEST improved with training from mean (sd): 15.14 (6.01) to 18.14 (6.34); *p* < 0.022	Harm: No falls or MSK injuries reported
Schlenstedt et al., (2018) [47] **(Dual-arm)**	RCT (post-hoc analysis of PD-FOG+ only) Quality: Good (6/10; PEDro scale)	PD + FOG with postural instability (total *n* = 20) Resistance group: *n* = 12 Balance group: *n* = 8	RT (Resistance Training): LE muscle exercises with bodyweight, cuff weights, elastic bands BT (Balance Training): Static and dynamic postural control tasks (e.g. reactive balance, weight shifting)	Training was 7 weeks, 2×/week for 60 min. Per session Group training (4–5 people). Unclear who delivered the intervention.	Motor Domain, Fullerton Advanced Balance Scale (FAB)	RT: FAB increased from mean (sd): 21.1 (4.7) to 23.2 (5.0); *p* = 0.245 BT: FAB increased from 22.4 (5.2) to 22.4 (5.7); *p* = 1.00	None reported
Scully et al., (2021) [48]	Double-blind (pilot) RCT Quality: Good (6/10; PEDro scale)	PD + FOG (*n* = 10 in active group)	The intervention consisted of individual psychobehavioral intervention (PBI) with group PT afterwards	Intervention was 4 weekly sessions, 1-h sessions, with an hour of group PT after	Limbic Domain, State-Trait Anxiety Inventory; STAI (Trait subscore)	The PBI group demonstrated an increase (worsening) in the STAI-Trait following intervention, with group means (sd): 44.70 (4.11) to 46.40 (5.54) These changes did not reach statistical significance.	None reported
Silva-Bautista et al., (2020) [50] **(Dual-arm)**	Parallel-group, single blind RCT (2 active participant groups) Quality: Good (6/10; PEDro checklist)	PD + FOG (*n* = 32) Resistance training + instability (ARTI) *n* = 17 Traditional motor rehabilitation (TMR) *n* = 15	Progressive program, consisting of either: ARTI: 7 whole-body free weight exercises performed on unstable surfaces (e.g. foam pad, BOSU, etc.) TMR: Gait, balance, posture stretching, & whole-body free-weight exercises	Training was performed 3×/week for 12 weeks (36 training sessions); 80-90 min. Each ARTI sessions were individualized and monitored by trainers TMR: group-based (up to 8 individuals) monitored by a physical therapist	Cognitive Domain: Stroop Color-Word Test (cognitive interference)	ARTI: The Stroop score improved (decreased) from mean +/− sd: 79.1 (a.u.) +/− 40.3 to 65.1 +/− 31.4. *p* < 0.0001 TMR: The Stroop score improved (decreased) from mean +/− sd: 72.1 (a.u.) +/− 44.4 to 72.5 +/− 41.1. *p* = 0.997	Harm: 1 participant experienced “lower-limb” pain in the ARTI group & dropped out; 2 participants (1 in each group) had injurious falls off-site
Walton et al., (2018) [54]	Double-blind RCT (active vs control group) Subset of data analyzed for those exhibiting FOG with baseline testing Quality: Good (6/10; PEDro scale)	PD + FOG (*n* = 18 in active group)	Computerized, progressive cognitive training Training consisted of psychoeducation on relevant PD topics then cognitive training with focus on set shifting, inhibition, memory, processing speed, etc.	Training consisted of 2-h sessions, 2×/week for 7 weeks (14 total sessions) Group format (10 or less participants); supervised by study authors	Cognitive Domain: Trail-Making-Test Part B (TMT-B) (s) Limbic Domain; Hospital Anxiety & Depression Scale (HADS)	The TMT-B time decreased from geometric mean (95% CI): 136.08 (110.52, 167.51) to 122 (99.12, 150.29). The HADS decreased (improved) from geometric mean (95% CI): 12.70 (9.92, 15.48) to 11.50 (8.72, 14.28). Neither result was statistically significant compared to control.	None reported

Abbreviations: PD + FOG: Individuals with Parkinson's Disease (PD) and Freezing of Gait (FOG); RCT: randomized controlled trial; sd: standard deviation; MiniBEST: Mini Balance Evaluation Systems Test; SEM: standard error of the mean; RAS: rhythmic auditory stimulation; PT: Physical therapist or Physical therapy; MSK: musculoskeletal; CI: confidence interval; PEDRO: Physiotherapy Evidence Database; STROBE: Strengthening the Reporting of Observational Studies in Epidemiology.

Many of the included groups received motor domain interventions, such as gait and balance training (e.g., multidirectional weight shifts, repeated pull tests, and/or walking on unstable surfaces) [6], [37], [47], or lower-body resistance exercise [2], [47]. Other programs combined cognitive and motor domain training, such as dual-tasking (e.g., naming items, counting) with gait training, boxing, tai-chi, etc. [8], [29]. Multiple studies utilized action observation training [1], [41] or skilled treadmill gait training utilizing virtual reality [4] or external cueing with biofeedback [19]. A few studies featured a multidisciplinary program incorporating motor, cognitive, and limbic interventions, such as complex gait and balance training, cardiovascular exercise, goal-setting, and/or psychoeducational therapy [10], [45].

Intervention schedules varied. Most programs lasted 6 weeks, with a range of frequencies (e.g., 1.5 h/week [45], 2×/week [8], 3×/week [4], [29], and 18 sessions without frequency provided [37]). Some studies lasted either 4–5 weeks [1], [6], [10], [19] 7 weeks [47], or 6 months [2]. Two studies [10], [45] featured intensive designs, such as a “FOG bootcamp” or short-stay inpatient format [10]. One study featured a multicenter fall prevention program at home [2]. Programs ranged from individualized delivery to small groups; some studies did not specify delivery (Table 1).

Outcomes assessing postural control varied across studies, with five studies using the Mini-Balance Evaluation Systems Test (MiniBEST) [2], [4], [6], [29], [45] and five the Berg Balance Scale (BBS) [1], [8], [10], [19], [41]. Others used the Fullerton Advanced Balance (FAB) [47] or the Gait and Balance Scale Part B (GABS-B) [37]. All studies trended toward a positive training effect on postural control following rehabilitation, though reached statistical significance for only 6 of the 17 participant groups [6], [10], [19], [41].

#### 3.2.2 FOG-related cognitive domain

Five studies provided within-group data reflecting executive functioning following rehabilitation and exercise in PD + FOG. Of these, three studies [4], [36], [50] included useable data from two independent, intervention arms. Thus, 8 unique participant groups and interventions were included, totaling 193 individuals with PD + FOG (Table 1).

Interventions largely utilized gait training or other task practice with or without dual-tasking exercise [29], virtual reality obstacle training [4], or progressive resistance exercise with instability [4], [29]. One program [36] utilized action observation training with or without sonification and one intervention [54] used computerized cognitive training targeting inhibitory control, attentional set-shifting, working memory, processing speed and visuospatial skills, in addition to PD-specific education. Training varied from 3×/week over 6 weeks [4], [29], 2×/week for 7–8 weeks [36], [54], and 3×/week for 12 weeks [50].

Outcomes included Stroop color and word test metrics [29], [50] or the Trail-Making-Test Part B (TMT—B) [4], [36], [54], considered to reflect attentional set-shifting and processing speed in individuals with PD [33]. One study [36] provided both TMT-B times and Stroop times; the TMT-B data were extracted for meta-analysis for greater homogeneity with metrics extracted from other studies. All but two groups trended toward improvement in cognition following training. None of the effects reached statistical significance in individual studies.

#### 3.2.3 FOG-related limbic domain

Four studies [5], [42], [48], [54] provided within-group data on anxiety and/or depression outcomes, totaling 55 PD + FOG (Table 1).

Interventions included computerized cognitive training [54] and progressive motor learning training with FOG-provoking situations and functional task practice [42]. Two studies utilized psychobehavioral therapy before group rehabilitation [48] or cognitive behavioral therapy consisting of thought records, mindfulness, and skills practice [5]. Intervention schedules spanned 1×/week for 4 weeks [5], [48], 3×/week for 6 weeks [42], or 2×/week for 7 weeks [54].

Outcomes included the State-Trait Anxiety Inventory (STAI)-Trait subscore [48], the Hospital Anxiety and Depression Score [54], the Depression Anxiety Stress Scale-21 (DASS-21)-Anxiety subscore [5] and the Geriatric Depression Scale [42]. Results were mixed, as two studies trended toward improvements following treatment [5], [54] and two reported worsening [42], [48]. None of the individual study effects reached statistical significance.

### 3.3 Risk of bias

Eleven studies [1], [2], [4], [6], [8], [10], [36], [47], [48], [50], [54] were rated as “Good” (6–8/10) on the PEDro scale. Two studies [29], [41] scored “Fair” on the PEDro scale. Of the five studies for which the STROBE checklist was used, two [5], [45] were rated as “excellent” (20/22, 91% and 19/21, 90%), two [37], [42] “good” (15–16/21, 71–75%), and one [19] “fair” (14/22, 64%) (Table 1; Supplemental Material #2).

### 3.4 Synthesis of results

#### 3.4.1 FOG-related motor domain

**Fig. 2:**
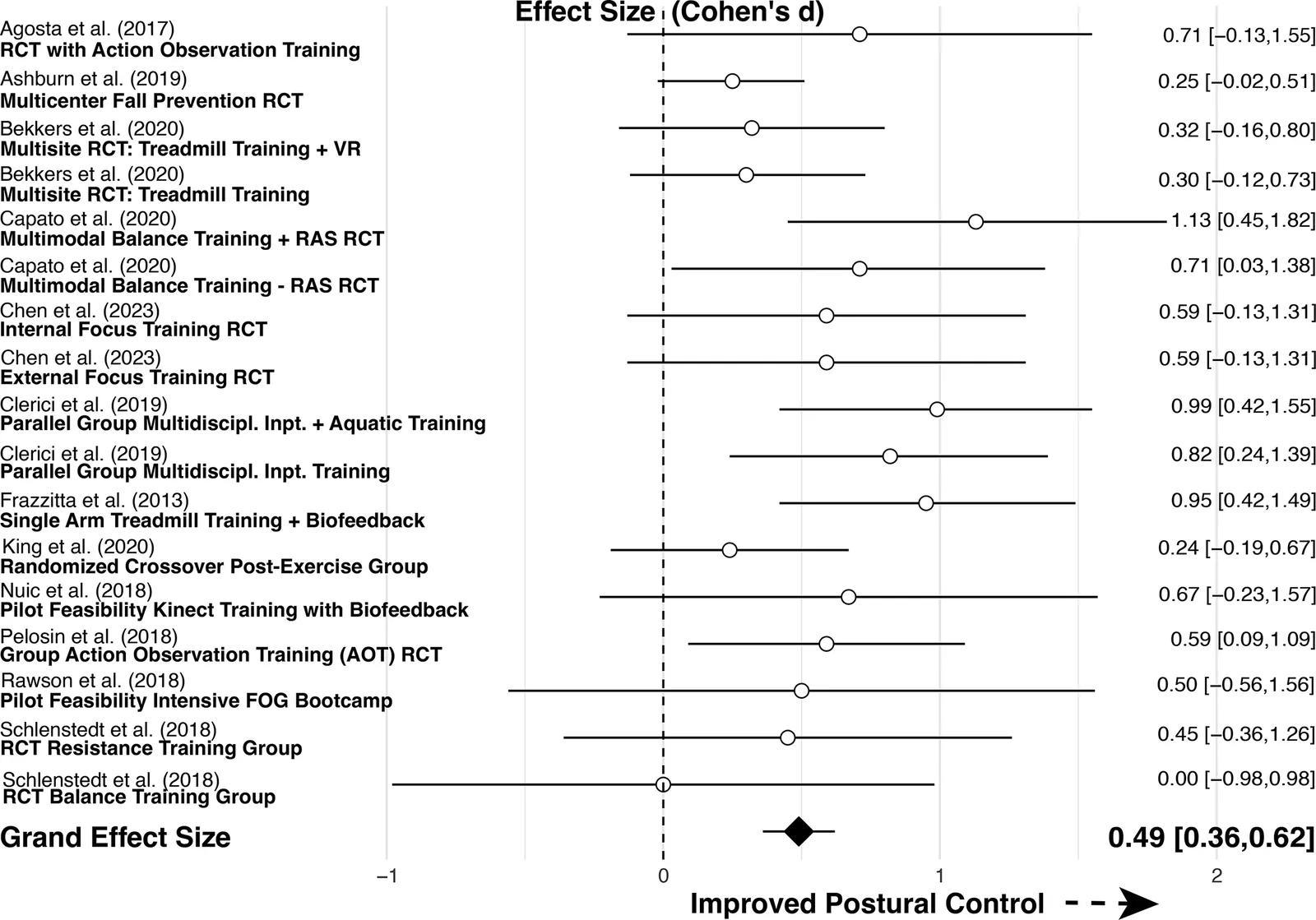
FOG-related motor domain forest plot reflecting postural control in individuals with Parkinson's disease and freezing of gait. Abbreviations: RCT = randomized controlled trial, VR = virtual reality, RAS = rhythmical auditory stimuli, Inpt. = inpatient, FOG = freezing of gait.

We performed the meta-analysis for postural control using a fixed effect model (Q = 18.31, *p* = 0.31). Our results suggest that rehabilitation was associated with a moderate improvement in postural control which was statistically significant: d = 0.49 (0.36, 0.62) (Fig. 2).

To convert this effect size to an interpretable clinical measure, we chose the MiniBEST as used in Rawson et al. [45] to represent our results. A grand effect size of d = 0.49 suggests that the MiniBEST might improve by ∼3 points following treatment. Three points is below the published minimal clinically important difference (MCID) of 3.4–4.0 [22] as well as the minimum detectable change at the 95% confidence level (MDC(95)) of 3.5 points [23] for individuals with PD.

#### 3.4.2 FOG-related cognitive domain

**Fig. 3:**
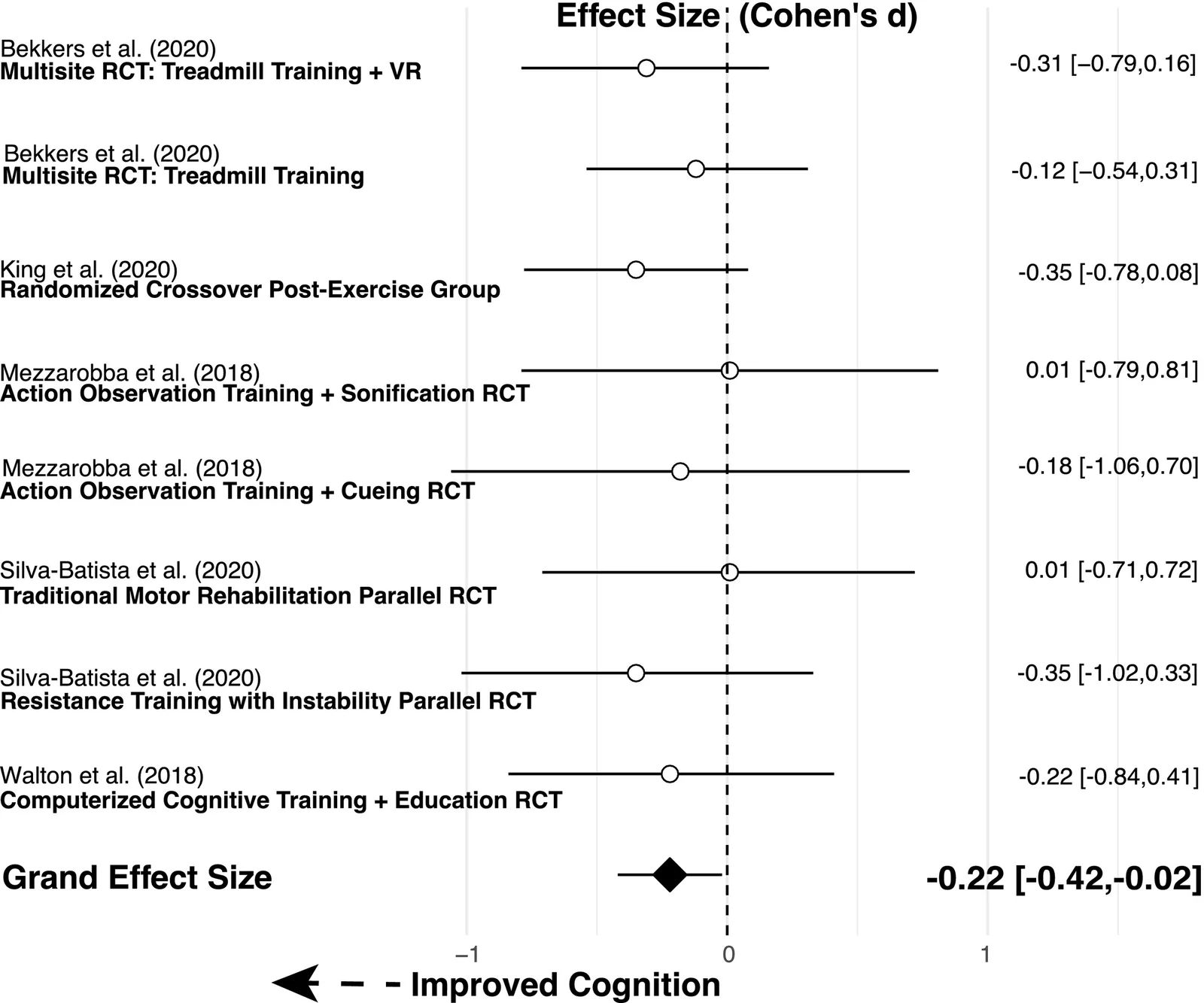
FOG-related cognitive domain forest plot reflecting attentional set-shifting and processing speed in individuals with Parkinson's disease and freezing of gait. Abbreviations: RCT = randomized controlled trial, VR = virtual reality.

A fixed effect model was used for meta-analysis of cognitive functioning (Q = 1.58, *p* = 0.98). The results suggest that rehabilitation was associated with a small, statistically significant pre-post improvement in cognitive function: d = −0.22 (−0.42, −0.02) (Fig. 3).

To convert this effect size to a clinical measure, we chose the TMT-B as used by Bekkers et al. [4] to represent our results. A grand effect size of d = −0.22 suggests that patients might take 15.4 fewer seconds to complete the TMT-B test following treatment, (∼10% less time), however, no MCID or MDC exists in PD for this test.

#### 3.4.3 FOG-related limbic domain

**Fig. 4:**
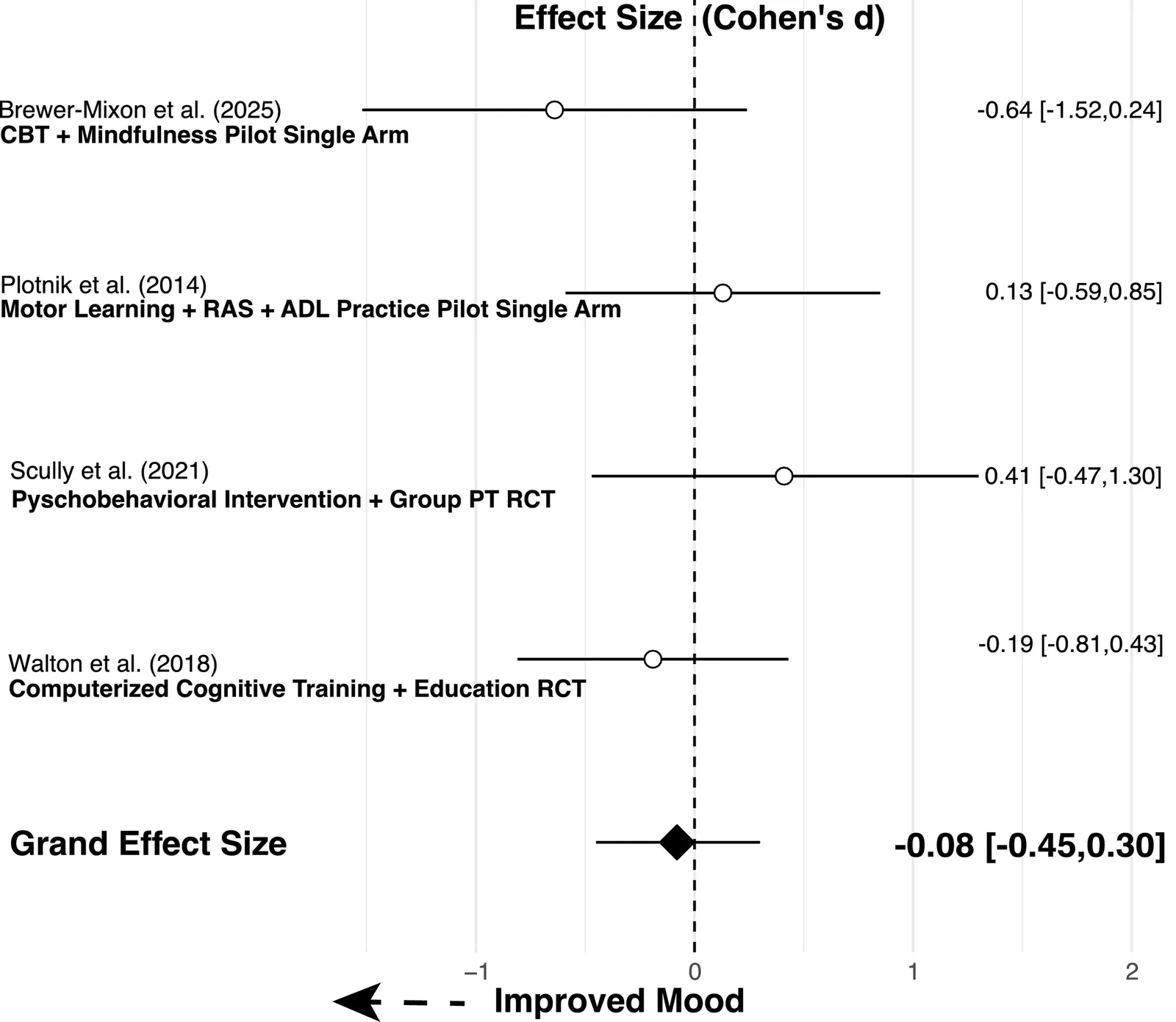
FOG-related limbic domain forest plot reflecting anxiety and/or depression in individuals with Parkinson's disease and freezing of gait. Abbreviations: RCT = randomized controlled trial, CBT = cognitive behavioral therapy, RAS = rhythmical auditory stimuli, ADL = activities of daily living, PT = physical therapy.

A fixed effect model was used for meta-analysis of limbic functioning (Q = 3.20, *p* = 0.36). Our results suggest a very small, nonsignificant treatment-related effect for anxiety and/or depression: d = −0.08 (−0.45, 0.30) (Fig. 4).

To convert this effect size to a clinical measure, we chose the HADS as used by Walton et al. [54] to represent our results. A grand effect size of d = −0.08 suggests a negligible improvement on the HADS total of 0.51 points. This number is below a reported MCID for HADS-total score of ∼1.60 in individuals with chronic obstructive pulmonary disease undergoing respiratory rehabilitation [43]; an MCID is not yet established for PD.

### 3.5 Harm or risk

Nine studies [2], [8], [10], [29], [36], [37], [42], [45] reported harm or risk associated with rehabilitation, with variable reporting. Six studies [2], [8], [36], [37], [42], [45] reported adverse events, with most deemed training-unrelated. For one study [29], adverse events included one fall resulting in hip fracture during class, and 4 non-injurious falls (Table 1).

### 3.6 Cost

Two studies [2], [10] mentioned cost, with Ashburn et al. [2] concluding that their fall prevention intervention was not cost-effective for PD + FOG. Clerici et al. [10] commented that the utilization of an inpatient setting to deliver treatment may be cost-prohibitive (Table 1).

## 4 Discussion

### 4.1 Summary of findings

Our results suggest that existing rehabilitation approaches moderately improve motor (postural control) systems and to a lesser extent, cognitive (executive function) systems in PD + FOG. The limbic system was minimally changed through intervention. Converted estimates of our results suggest none of the observed changes exceeded established MCID thresholds; however, interpretation was limited by the lack of validated MCID/MDC values for this population for the cognitive and limbic domains.

### 4.2 Interpretation of findings

#### 4.2.1 FOG-related motor domain

The gait and balance training most frequently used in PD + FOG rehabilitation is hypothesized to target the dysfunctional integration of sensorimotor neural circuits spanning cortical and subcortical regions contributing to FOG through replication of task-specific balance, timing, and amplitude demands to improve motor automaticity and develop compensatory strategies [30]. Our studies featured several strategies associated with improved postural control in PD + FOG, such as action observation training, balancing on unstable surfaces, and gait training with external cueing. While the resulting effect size did not reach clinical importance, some participants (such as the 155 individuals scored on the BBS) crossed over from being classified as “fallers” to “non-fallers,” supporting a meaningful change in functional status.

Notably, these postural control outcomes for PD + FOG were not as robust as literature suggests for those with less-advanced PD. The CPG for treating PD that provided strong support for various motor-focused rehabilitative methods relied on “A high level of certainty of moderate-to-substantial benefit” [39]. Our results in PD + FOG showed a smaller benefit, possibly because of the symptom's multi-domain pathophysiology resulting in incomplete alleviation with motor-focused treatment alone. Interestingly, our meta-analysis suggests that higher effect sizes are not consistently related to higher frequency or intensity of rehabilitation sessions. For example, both groups eligible for analysis from Capato et al. [6] received the same frequency and intensity (2×/week for 5 weeks) of multimodal balance training, but the groups were randomized to train with (effect size 1.13, considered large) or without (effect size 0.71, considered moderate) rhythmic auditory stimulus cueing. Capato et al. [6] reported a statistically significant difference in improvement between these two groups post-intervention. Our results show that the effect sizes across our studies trend slightly higher in groups that had combined motor plus non-motor or cognitively challenging training, suggesting that future rehabilitation may benefit from similar protocols.

While our results support a benefit in postural control, few studies reported longer-term treatment outcomes. One exception is Capato et al. [6] who reported that their group receiving multimodal balance training with rhythmic auditory stimulus cueing retained improvements over baseline at 1-month and 6-month follow-ups. The durability of motor effects from other interventions for PD + FOG requires additional research.

#### 4.2.2 FOG-related cognitive domain

Our results suggest that incorporating cognitive training as part of comprehensive rehabilitation may be associated with small (∼10%) improvements in attentional set-shifting and processing speed in PD + FOG. It is hypothesized that interventions such as dual-tasking and agility practice, VR-based obstacle navigation, and computerized cognitive training act on dysfunctional frontal-basal ganglia circuits governing set-shifting, attention, and response inhibition in this population [13], [38]. Since FOG often occurs during motor task switching (e.g., gait initiation or from walking to turning), cognitive rehabilitation may improve one's ability to process and integrate sensory information to restore movement automaticity or stabilize motor output.

No individual studies examining cognitive processing showed statistically significant effect sizes, perhaps because of small sample sizes (*n* = 10 to 43) and wide confidence intervals; however, our meta-analysis allowed pooling of data to reveal a significant but small effect. Heterogeneity across intervention schedules likely also contributed to the effect size (e.g., total intervention times varied from 13.5 [4] to 54 h [50]). However, effects appeared to depend more on the addition of cognitive load in the interventions, as Silva-Batista et al. [50] showed effect sizes of 0.01 (−0.71, 0.72) following traditional resistance exercise in a participant group versus −0.35 (−1.02, 0.33) indicating less cognitive interference after resistance exercise performed on unstable surfaces in another group. Both groups had the same treatment time of 54 h over the same duration of 12 weeks. Furthermore, computer-based cognitive training [54] and more dynamic activities (e.g., VR treadmill training [4]) both exhibited similar trends in improvements, suggesting patients and clinicians may have options when selecting training based on access, safety, and relevance. As only five studies examined these outcomes, the optimal format and parameters for training remains unclear.

#### 4.2.3 FOG-related limbic domain

Our results revealed that anxiety and depression were not consistently improved through rehabilitation. We hypothesize that these limited and inconsistent effects may reflect both the small number of studies (four) that measured limbic symptoms and also variability in intervention specificity.

The amygdala is a chief limbic structure linked to threat perception and may compete with cognitive processing during motor responses [20]. FOG is associated with altered connectivity between the amygdala and putamen and frontoparietal regions mediating attention [20], suggesting a potential mechanism for the effects anxiety and fear may have in motor breakdown. Limited research has examined limbic system interventions in PD + FOG which are designed to directly address these impairments, with general cognitive behavioral therapy (CBT) found to worsen anxiety [9], [48], and more individualized treatment (such as mindfulness-based CBT and FOG-specific skills practice [5]) improving limbic symptoms. Further research is needed to determine which limbic symptoms, such as depression, anxiety, or stress, are most relevant to FOG and which interventions best address these symptoms or their interaction with other features of FOG.

Together, our findings suggest a mismatch between the characterization of FOG as a product of interacting, dysfunctional multi-domain neural systems and its current and historic assessment and treatment focusing on addressing related motor impairments. Therefore, this mechanistic mismatch is thought to result in incomplete system-level intervention and a difficulty in achieving meaningful treatment benefit despite motor gains using rehabilitation and exercise. It is expected that enhancing the capacity of rehabilitation clinicians to assess and address both motor and non-motor FOG-related impairments may facilitate improved care coordination and reduced symptom burden in PD + FOG.

While rehabilitation was associated with changes across the FOG-related motor and cognitive symptom domains, the causal relationships between these motor and non-motor symptom changes and corresponding improvements in FOG remain unclear. Specifically, it cannot be determined whether improvements in postural control, cognition, and limbic function contribute to reductions in FOG severity, result from improvements in FOG, or represent independent responses altogether. Future studies incorporating participant-level data examining these relationships, as well as stratification by FOG phenotype, are necessary to better understand these complex relationships and the mechanisms underlying rehabilitation response for PD + FOG.

### 4.3 Strength of recommendations/GRADE

Fifteen studies were rated as “good” or “excellent” on the PEDro or STROBE measures, while three studies were “fair,” suggesting a small risk of bias in the studies examined. For the motor and cognitive FOG-related symptom domains, point estimates and confidence intervals were relatively similar across studies, suggesting limited data inconsistency. However, for the limbic FOG-related symptom domain, the point estimates and confidence intervals were largely split in their directionality, suggesting considerable inconsistency. Furthermore, for the domains examined, the postural control outcomes synthesized were largely similar, suggesting greater directness in their assessment. Various outcome measures were used for the cognitive and limbic domains, however, suggesting potential indirectness for these results. Thus, we have greater confidence in the estimated effects for the motor domain and somewhat less for the cognitive domain. Evidence for the limbic domain remains quite limited and uncertain.

### 4.4 Limitations

Primary limitations include the use of only two research databases, which may have omitted relevant studies due to the search strategy. To partially mitigate this limitation, a later search was performed (July 2026) in Embase using the same strategy as before. Screening of 834 related records yielded no additional studies that met the inclusion criteria. Limitations regarding data analysis may include the heterogeneity (particularly among cognitive and limbic domains) for the outcome measures selected and combined in the domain-specific meta-analyses. Further, limitations may also stem from data extraction, which required estimations (e.g., from boxplots) or conversions to mean and standard deviation performed in accordance with published methods, potentially introducing error. Lastly, our results may also be influenced by participant factors such as cognitive status and disease severity or duration; however, we were unable to perform subgroup or meta-regression analyses examining these influences due to substantial heterogeneity across the included studies in the stratification and reporting of these outcomes.

### 4.5 Directions for future research

Future research is needed to develop and test multidomain rehabilitation interventions that shift from motor-dominant rehabilitation to mechanism-informed strategies which account for and address a client's predominant domain impairments. For example, a future workflow for clinicians could utilize a tool (e.g., the C-FOGQ) which categorizes and assesses the relative motor, cognitive, and limbic domain contributors to the client's FOG, then an integrated, comprehensive intervention is implemented which is similarly biased in its components. Similarly, rehabilitation-based studies must also begin to utilize FOG subtyping or stratification for further elucidating potential differential treatment responses. While further research is needed, we strongly suggest that sufficient data exists to begin to add cognitive and limbic interventions such as dual-task training, task switching, and FOG-specific CBT or mindfulness training to existing rehabilitation programs to optimize relevance and benefit. Additional research should prioritize the investigation of optimal dosing parameters (e.g., frequency and intensity) of combined treatment approaches, as well as whether rehabilitation duration independently influences treatment outcomes.

Multiple studies in our meta-analysis featured a short-stay, intensive inpatient setting or “bootcamp” format, which may limit scalability due to cost and operational constraints. Intensive exercise performed with cognitive load and aspects of CBT may still be possible in group settings, telerehabilitation, or other models at slightly lower cost. In addition, continued mechanistic research is needed to further characterize the presence and prevalence of multidomain impairments in PD + FOG, thereby guiding the development of ongoing targeted rehabilitation strategies and assessment tools.

## 5 Conclusion

We found that rehabilitation was associated with significant, moderate improvements in postural control (motor domain), smaller, significant improvements in attentional set-shifting and processing speed (cognitive domain), and minimal, nonsignificant effects in depression and anxiety (limbic domain) for PD + FOG. Our results suggest that rehabilitation strongly engages motor circuits, somewhat engages cognitive circuits, and minimally engages limbic circuits, likely contributing to a lack of complete or meaningful FOG resolution for this population. Future research and clinical practice should assess multidomain impairments in PD + FOG and work to develop, test, and implement multidomain assessment and treatment strategies.

## CRediT authorship contribution statement

**Jessica E. Bath:** Writing – review & editing, Writing – original draft, Visualization, Methodology, Investigation, Formal analysis, Conceptualization. **Diane D. Allen:** Writing – review & editing, Validation, Methodology, Formal analysis. **Valerie J. Block:** Writing – review & editing, Validation, Supervision, Formal analysis.

## Disclosure statement

No competing interests to disclose.

## Funding

No funding sources.

## Declaration of competing interest

The authors declare the following financial interests/personal relationships which may be considered as potential competing interests: Jessica Bath reports a relationship with Skip Movewear that includes: consulting or advisory. If there are other authors, they declare that they have no known competing financial interests or personal relationships that could have appeared to influence the work reported in this paper.
