# Supplementary material for: Motor and non-motor outcomes post rehabilitation for persons with freezing of gait and Parkinson's disease: A systematic review and Meta-analysis
Source: Clin Park Relat Disord. 2026 Sep 7;15:100498. doi: 10.1016/j.prdoa.2026.100498 (PMC13599663; doi:10.1016/j.prdoa.2026.100498)
Supplement: Supplementary file 1 [file mmc1.docx]

**Supplemental Material #1: Full Search Strategy and Journals Searched**

1) **CINAHL**

(MH "Parkinson Disease+") OR "Parkinson’s disease" OR "Parkinsons disease" OR PD AND ("freezing of gait" OR FOG OR freezer* OR "gait freezing" OR "FOG-Q" OR "NFOG-Q")

AND (intervention* OR rehabilitation OR "physical therapy" OR physiotherapy OR exercise OR training OR therapy OR program OR protocol OR "cognitive training" OR "dual task" OR "motor training" OR "action observation" OR AOT OR "balance training" OR mindfulness OR yoga OR meditation OR "cognitive behavioral therapy" OR CBT OR "mind-body" OR qigong OR "tai chi" OR "virtual reality" OR VR OR cueing)

AND (anxiety OR anxious OR "fear of falling" OR "GAD-7" OR HADS-A OR affective OR limbic OR mood OR depression OR cognit* OR "executive function" OR attention OR "set shifting" OR "task switching" OR "TMT-B" OR Stroop OR "dual task cost" OR "working memory" OR gait OR walking OR balance OR mobility OR "postural control" OR "Timed Up and Go" OR TUG OR MiniBESTest OR asymmetry OR variability OR "gait speed" OR turning)

2) **PubMed**

("Parkinson Disease"[Mesh] OR "Parkinson’s disease" OR "Parkinsons disease"

OR PD)

AND ("Freezing of gait" OR FOG OR freezer* OR "gait freezing" OR "FOG-Q" OR "NFOG-Q")

AND (intervention* OR rehabilitation OR "physical therapy" OR physiotherapy OR exercise OR training OR therapy OR program OR protocol OR "cognitive training" OR "dual task" OR "motor training" OR "action observation" OR AOT OR "balance training" OR mindfulness OR yoga OR meditation OR "CBT" OR "cognitive behavioral therapy" OR "mind-body" OR Qigong OR Tai Chi OR "virtual reality" OR VR OR cueing)

AND (anxiety OR anxious OR "fear of falling" OR "GAD-7" OR "HADS-A" OR affective OR limbic OR mood OR depression OR cognit* OR "executive function" OR attention OR "set shifting" OR "task switching" OR "TMT-B" OR Stroop OR "dual task cost" OR "working memory" OR gait OR walking OR balance OR mobility OR "postural control" OR "Timed Up and Go" OR TUG OR "MiniBESTest" OR asymmetry OR variability OR "gait speed" OR turning)

**Supplemental Material #2a-2b: Study Quality**

**Table 2a: Study Quality for Randomized Controlled Trials using the Physiotherapy Evidence Database Scale**

| **PEDro Scale** | **Agosta et al., (2017)[****1****]** | **Ashburn et al., (2019)[****2****]** | **Bekkers et al., (2020) [****4****]** | **Capato**  **et al.,  (2020) [****6****]** | **Chen**  **et al.,**  **(2023)[****8****]** | **Clerici**  **et al.,**  **(2019) [****10****]** | **King**  **et al.,**  **(2020) [****29****]** | **Mezzarobba et al., (2018)[****36****]** | **Pelosin et al., (2018) [****41****]** | **Schlenstedt et al.,**  **(2018)[****47****]** | **Scully**  **et al.**  **(2021) [****48****]** | **Silva-Batista et al., (2020) [****50****]** | **Walton et al., (2018) [****54****]** |
| --- | --- | --- | --- | --- | --- | --- | --- | --- | --- | --- | --- | --- | --- |
| *1. Eligibility criteria were specified (not included in final score)* | *Y* | *Y* | *Y* | *Y* | *Y* | *Y* | *Y* | *Y* | *Y* | *N* | *Y* | *Y* | *Y* |
| 2. Subjects were randomly allocated to groups (in a crossover study, subjects were randomly allocated an order in which treatments were received) | Y | Y | Y | Y | Y | Y | Y | Y | Y | Y | Y | Y | Y |
| 3. Allocation was concealed | Y | Y | N | Y | Y | Y | N | Y | N | N | N* | N* | N* |
| 4. The groups were similar at baseline regarding the most important prognostic indicators | Y | N | Y | Y | Y | Y | N | Y | Y | Y | Y | Y | N |
| 5. There was blinding of all subjects | N | N | N | N | N | N | N | N | N | N | N | N | N |
| 6. There was blinding of all therapists who administered the therapy | N | N | N | N | N | N | N | N | N | N | N | N | N |
| 7. There was blinding of all assessors who measured at least one key outcome | Y | Y | Y | Y | Y | Y | Y | Y | N | Y | Y | Y | Y |
| 8. Measures of at least one key outcome were obtained from >85% of the subjects initially allocated to groups | N | Y | Y | Y | Y | Y | N | Y | Y | N | Y | N | Y |
| 9. All subjects for whom outcome measures were available received the treatment or control condition as allocated or, where this was not the case, data for at least one key outcome was analyzed by “intention to treat” | N | Y | N | Y | N | N | N | N | N | Y | N | N | N |
| 10. The results of between-group statistical comparisons are reported for at least one key outcome | Y | Y | Y | Y | Y | Y | Y | Y | Y | Y | Y | Y | Y |
| 11. The study provides both point measures and measures of variability for at least one key outcome | Y | Y | Y | Y | Y | Y | Y | Y | Y | Y | Y | Y | Y |
| **Score Total & Quality (/10)** | **6/10 (Good)** | **7/10 (Good)** | **6/10**  **(Good)** | **8/10**  **(Good)** | **7/10**  **(Good)** | **7/10**  **(Good)** | **4/10**  **(Fair)** | **7/10**  **(Good)** | **5/10**  **(Fair)** | **6/10**  **(Good)** | **6/10**  **(Good)** | **6/10**  **(Good)** | **6/10**  **(Good)** |

**Table 2b: Study Quality for non-Randomized Controlled Trials using the Strengthening the Reporting of Observational Studies in Epidemiology (STROBE) checklist**

| **STROBE Checklist Item** | **Brewer Mixon**  **et al.,**  **(2025)[****5****]** | **Frazzitta**  **et al.,**  **(2013)[****19****]** | | **Nuic et al.,**  **(2018)[****37****]** | | **Plotnik et al.**  **(2014)[****42****]** | | **Rawson et al.,**  **(2018)[****45****]** | |  |
| --- | --- | --- | --- | --- | --- | --- | --- | --- | --- | --- |
| 1. Title and abstract | Y | N | | Y | | N | | *Y* | |  |
| 2. Background/rationale | Y | Y | | Y | | Y | | Y | |  |
| 3. Objectives | Y | Y | | Y | | Y | | Y | |  |
| 4. Study Design | Y | Y | | Y | | Y | | Y | |  |
| 5. Setting | Y | Y | | Y | | Y | | Y | |  |
| 6. Participants | Y | Y | | Y | | Y | | Y | |  |
| 7. Variables | Y | Y | | Y | | Y | | Y | |  |
| 8.Data sources/measurement | Y | Y | | Y | | Y | | Y | |  |
| 9. Bias | Y | N | | N | | N | | Y | |  |
| 10. Study size | N | N | | N | | N | | N | |  |
| 11. Quantitative variables | Y | Y | | Y | | Y | | Y | |  |
| 12. Statistical methods | N | N | | N | | N | | N | |  |
| 13. Participants | Y | N | | N | | Y | | Y | |  |
| 14. Descriptive data | Y | N | | Y | | N | | Y | |  |
| 15. Outcomes data | Y | Y | | Y | | Y | | Y | |  |
| 16. Main results | Y | | Y | | Y | | Y | | Y | |
| 17. Other analyses | Y | | Y | | n/a | | n/a | | n/a | |
| 18. Key results | Y | | Y | | Y | | Y | | Y | |
| 19. Limitations | Y | | Y | | Y | | Y | | Y | |
| 20. Interpretation | Y | | Y | | Y | | Y | | Y | |
| 21. Generalizability | Y | | N | | N | | Y | | Y | |
| 22. Funding | Y | | N | | Y | | N | | Y | |
| **Score Total & Quality (/# items scored)** | **20/22, 91%**  **(Excellent)** | | **14/22, 64%**  **(Fair)** | | **16/21, 76%**  **(Good)** | | **15/21, 71%**  **(Good)** | | **19/21, 90%**  **(Excellent)** | |
